# Does Radial Extracorporeal Shockwave Therapy Applied to the Achilles Tendon Influence Ankle Functionality?

**DOI:** 10.3390/jfmk9020067

**Published:** 2024-04-08

**Authors:** Younglan Joo, Wonjae Choi, Jihye Jung, Hyunjoong Kim, Sungeon Park, Sangbong Lee, Seungwon Lee

**Affiliations:** 1 Minimal Pilates & Move, PH 24, UN Village-gil, Yongsan-gu, Seoul 04420, Republic of Korea; joojoo@minimalpilates.com; 2 Department of Physical Therapy, Joongbu University, 201, Daehak-ro, Chubu-myeon, Geumsan 32713, Republic of Korea; wjchoi@joongbu.ac.kr; 3 Institute of SMART Rehabilitation, Sahmyook University, 815, Hwarang-ro, Nowon-gu, Seoul 01795, Republic of Korea; jihye3752@gmail.com; 4 Department of Senior Exercise Prescription, Gwangju Health University, 73, Bungmun-daero 419beon-gil, Gwangju 62287, Republic of Korea; hyun-joongkim@nmslab.org; 5 Department of Physical Therapy, Graduate School, Sahmyook University, 815, Hwarang-ro, Seoul 01795, Republic of Korea; spring1056@naver.com (S.P.); leesb109@naver.com (S.L.); 6 Department of Physical Therapy, Sahmyook University, 815, Hwarang-ro, Seoul 01795, Republic of Korea

**Keywords:** radial extracorporeal shockwave therapy, Achilles tendon, range of motion, physical therapy

## Abstract

This study aimed to determine the effectiveness of radial extracorporeal shockwave therapy (rESWT) in enhancing ankle function in patients with Achilles tendon injuries. The choice of rESWT was based on previous success in the treatment of musculoskeletal conditions. The study involved an intervention group that received rESWT, and a control group that received sham therapy. The results revealed that rESWT led to significant improvements in single-leg vertical jump (*d* = 0.55, *p* < 0.05), indicating enhanced power generation and ankle functionality that were not observed in the control group. Additionally, the therapy resulted in increased ankle mobility, as observed by improvements in plantar flexion and heel-rise tests. Interestingly, these functional gains were not accompanied by changes in the Achilles tendon stiffness, suggesting that the benefits of rESWT may be more functional than structural. This study highlights rESWT as a promising tool for rehabilitation, particularly following Achilles tendon injuries. The study concluded that, although rESWT appears to improve certain aspects of ankle function, further studies with a larger and more diverse population over a longer period are necessary to confirm these findings and establish comprehensive treatment protocols.

## 1. Introduction

Achilles tendon injury, a common ailment among athletes, is predominantly linked to dynamic, high-intensity activities involving sudden and repetitive movements [1]. These movements are particularly evident in a variety of sports, such as jumps or rapid toe push-offs, which are integral to sprinting, badminton, soccer, tennis, handball, basketball, and volleyball [2]. These sports, often performed in the Summer Olympics, are known for their intense physical demands, which unfortunately result in a high occurrence of Achilles tendon damage [3,4].

In-depth studies have revealed that structural changes in the Achilles tendon tissue are prevalent across different athletic disciplines. Approximately 25% of track athletes, 35% of athletes engaged in ball sports, and 26% of athletes participating in a range of other sports experienced these changes [5]. Interestingly, these injuries are more common in men (35%) than women (23%), with a marked increase in incidence among athletes aged >30 years, reaching as high as 50% [5]. The function of the Achilles tendon is vital; it not only provides elasticity during jumping but also facilitates a rapid stretch and recoil effect during landing, which is essential for athletic performance [4]. The term “tendonitis” for Achilles tendon issues is commonly used even though there is a lack of scientific evidence to indicate inflammation [6,7,8,9]. Reports suggest that the functional decline of the Achilles tendon is associated with tendinosis, which involves degenerative changes rather than inflammation [9,10,11]. This perspective is supported by the idea that physical activity is not the primary pathological cause but rather contributes to symptom development. Such degeneration at the cellular level, evidenced by altered tendon fiber structures and arrangements, as well as potential new blood vessel growth, is believed to result from cumulative microtrauma [9,10,12]. The limitations caused by this tendinosis limitation of ankle joint function due to the Achilles tendon manifests as reduced flexibility and impaired jumping and walking abilities, which are critical movements in many sports [13,14,15,16]. Age-related changes, including a decline in the fibrous and mucoid areas within the tendon and loss of collagen, further exacerbate the risk of tendon injury in older athletes [4].

Radial extracorporeal shock wave therapy (rESWT) has emerged as a notable solution for these issues in sports medicine and rehabilitation. Recognized for its effectiveness in restoring shortened muscles and normalizing fibrotic tissues in muscles or fascia, rESWT offers a promising therapeutic approach for softening fibrotic tissues and alleviating pain, thereby enhancing muscle and fascia relaxation [17,18]. In studies focusing on patients who have undergone actual tendon repair, it has been suggested that the application of rESWT to the myotendinous junction aids in promoting neovascularization and contributes to enhancements in muscle stiffness, thickness, and power [19]. This finding can partially explain the previously described increases in fibrous and mucoid content within the tendon, as well as the stimulation of collagen production. This therapy distinguishes itself from other methods because of its relatively short treatment duration and significant results it delivers, particularly in athletes [15]. Although existing studies primarily focus on the pain-relief effects of rESWT in treating conditions, such as plantar fasciitis and knee osteoarthritis [15,17], there is growing interest in understanding its functional impact, especially concerning ankle functionality, an area where studies are still developing.

This study aimed to investigate the functional impact of rESWT when applied to the Achilles tendon, with a specific focus on ankle functionality. Given the critical role of the Achilles tendon in facilitating various athletic movements and its susceptibility to injury, this study aimed to investigate whether rESWT, already established for its effectiveness in treating various musculoskeletal conditions, could also significantly improve the functional aspects of the ankle joint, particularly in athletes with tendon injuries. This exploration is crucial for advancing the understanding and treatment of Achilles tendon injuries in sports medicine.

## 2. Materials and Methods

### 2.1. Study Design

This was an accessor-blind, prospective, single-group intervention study structured in accordance with the guidelines of the Strengthening the Reporting of Observational Studies in Epidemiology (STROBE) Statement. This study aimed to enhance the clarity and reliability of the reporting of observational studies. This study was conducted between 26 and 30 December 2023 at Sahmyook University and Minimal Pilates (Seoul, Republic of Korea).

Fundamentally, this study involved a pretest to establish baseline data, followed by an examination of immediate post-intervention changes. The test design was a key characteristic of this study. The primary objective of this study was to analyze the direct effects of the intervention and provide critical information for practical application in the relevant field. By focusing on immediate post-intervention outcomes, this study aimed to provide significant insight into the efficacy of the intervention and its potential applications. 

### 2.2. Participants

The recruitment of participants for the study was planned at the Minimal Pilates center and Sahmyook University in Seoul, Republic of Korea. Potential participants selected through recruitment documents were enrolled upon meeting the eligibility criteria [20,21,22]. After a comprehensive explanation of the study was provided, those who voluntarily agreed to participate were recruited.

#### 2.2.1. Inclusion Criteria

- Individuals with ankle-related pain scores of 0–2 on the Numeric Pain Rating Scale (NPRS).
- Individuals with no functional impairment in the ankle.

#### 2.2.2. Exclusion Criteria

- Individuals who underwent surgical procedures, such as ankle joint arthrodesis.
- Individuals showing signs of functional impairment in ankle functionality.
- Individuals with ankle-related pain rated >3 on the NPRS.
- Individuals with acute ankle fractures.

### 2.3. Interventions

#### 2.3.1. Radial Extracorporeal Shockwave Therapy

We used the Masterpuls MP200 device (Storz Medical AG, Tägerwilen, Switzerland) to administer rESWT. During treatment sessions, the participants were positioned with their calves uncovered. To facilitate the effective transmission of shock waves, a coupling gel was applied to the area around the Achilles tendon. Shock waves were applied meticulously following the 90° application rule. We used a 15 mm applicator and moved it across the myotendinous junction of the Achilles tendon in both transverse and diagonal patterns. This approach ensures comprehensive coverage of the tendon and surrounding muscle tissue. The applicator was moved in a smooth and consistent manner to maximize the effectiveness of the therapy [19,23]. Each session delivered 1000 pulses at a frequency of 10 Hz and air pressure of 1.0 bar, which were parameters found to be effective in prior studies [17,24].

#### 2.3.2. Sham Extracorporeal Shockwave Therapy

A sham version of rESWT was used as a control [25]. In this sham intervention, we placed the rESWT machine on the participant, but we did not administer any shock waves. This approach was taken to ensure that the participants in the sham group had an experience similar to that of those receiving the actual rESWT, without therapeutic effects, adhering to the study ethics guidelines. This method allowed us to make a valid comparison between the effects of actual rESWT and a placebo-like condition, ensuring that any observed effects could be confidently attributed to the therapy itself.

### 2.4. Outcome Measures

#### 2.4.1. Primary Outcome Measure

The single-leg vertical jump was assessed using the OptoGait System (Microgate, S.R.L, Bolzano, Italy, 2010) to measure the maximum height, flight time, and ground contact time. Single-leg vertical jump performance was evaluated based on higher jump heights, longer flight times, and shorter ground contact times [26]. The set-up involved two parallel bars equipped with sensors placed on each side and a camera positioned in front. After removing their shoes, the participants stood between the bars. They were instructed to “please jump as high as possible, five times,” with the command being loudly given [27]. Data collected from these five jumps were processed using OptoGait software (Version 1.5.0.0, Microgate, S.R.L). Variables for the single-leg vertical jump were analyzed based on the average maximum height, flight time, and ground contact time extracted from the data. The test–retest reliability of the OptoJump system for this assessment was high, with an intraclass correlation coefficient (ICC) of 0.982–0.989, low coefficient of variation (2.7%), and minimal random error (±2.81 cm), making it suitable for evaluating vertical jump height [28].

#### 2.4.2. Secondary Outcome Measures

1. Achilles tendon stiffness

In this study, we specifically focused on assessing the stiffness of the Achilles tendon, which is a key factor in understanding its functional health. We used the Myoton PRO device (Myoton AS, Tallinn, Estonia), a sophisticated tool designed for the noninvasive evaluation of muscle and tendon properties, to assess Achilles tendon stiffness. The Myoton PRO is widely recognized for its ability to accurately measure muscle stiffness, which is defined as the resistance of a muscle tissue to external forces [29]. The reliability of the Myoton PRO in clinical assessments has been validated in previous studies, which reported high intra-rater reliability, with ICC values ranging between 0.94 and 0.99 [30]. During our assessment, the Myoton PRO probe was carefully positioned perpendicular to the myotendinous junction of the Achilles tendon. This alignment is crucial for obtaining accurate tendon stiffness readings. We obtained an average of three separate readings to ensure the precision of our measurements, as recommended in previous methodological studies [19].

2. Ankle joint range of motion

In this study, ankle joint mobility was precisely measured by evaluating both dorsiflexion and plantar flexion using a goniometer, specifically the Baseline Absolute-Axis Goniometer from HiRes, New York, NY, USA, 2008 [31]. This instrument is known for its accuracy in assessing joint angles. The reliability of the goniometer was supported by its high intra-observer reliability, with ICC values ranging between 0.91 and 0.99 [32].

3. Heel-rise

In this study, the heel-rise test was employed as a key indicator to predict the level of recovery after injury [33]. This test is particularly important for assessing the functional recovery of the lower extremities, especially after Achilles tendon injuries. To measure the height of the heel-rise, which is an important parameter for evaluating calf muscle strength and tendon function, we utilized the arm of a goniometer, a tool known for its precision in measuring angles and linear movements [33,34,35].

### 2.5. Ethics and Dissemination

This study was approved by the Institutional Review Board of Sahmyook University (No. 2-1040781-A-N-012021092HR) on 15 July 2021, prior to the registration of the first participant on 30 December 2023. This study was registered as a clinical trial at ClinicalTrials.gov (No. NCT06210152).

The participants were fully informed about the study, and written consent was obtained before proceeding with the study. The purpose and significance of the study, along with its procedures, were explained to the participants. Additionally, the potential risks or discomfort that may arise during the experiment, as well as plans for risk mitigation, were thoroughly discussed in writing. Subsequently, consent for participation was obtained from the participants on a voluntary basis.

The consent forms were drafted in layman’s terms, avoiding professional jargon to ensure ease of understanding for the participants. Furthermore, the confidentiality and anonymity of the participants’ personal information were strictly maintained. The researchers were able to answer any queries from the participants at any time, and the participants had the right to withdraw from the experiment at any stage.

### 2.6. Sample Size

We conducted a sample size calculation tailored for a single-group design. This was performed using a power calculator, specifically version 7.12 from the Institut Municipal d’Investigació Mèdica in Barcelona, Spain. The basis for this calculation was the observed standard deviation in changes in Achilles tendon length, as cited in a previous study in the literature [34]. Our calculations determined that a sample size of 26 randomly selected participants would provide the statistical power required to estimate the population mean reliably. This estimation was made with a 95% confidence level and aimed for a precision level of ±0.5 units, against a backdrop of a standard deviation of 1.3 units in the measured variable. By anticipating no need for participant replacement (0% replacement rate) and factoring in potential dropouts for robustness, we increased the total number of participants to 33.

### 2.7. Statistical Analysis

Statistical analysis was performed using SPSS version 23.0 for Mac, a choice driven by its comprehensive features and reliability in handling complex datasets. First, we assessed the distribution of our collected data using the Kolmogorov–Smirnov test. For the within-group analysis, particularly to understand the changes before and after the intervention, we employed a paired sample t-test. In cases where the data between different groups followed a normal distribution, an independent sample t-test was used. However, the Wilcoxon signed-rank test was used for variables that did not adhere to a normal distribution. To measure the magnitude of the effect of our intervention, we calculated the effect size for each variable using Cohen’s d. We categorized the results based on Cohen’s established criteria, defining small (*d* = 0.20), moderate (*d* = 0.50), and large (*d* = 0.80) effect sizes [36]. Finally, we set the threshold for statistical significance at α = 0.05.

## 3. Results

### 3.1. Characteristics of Enrolled Participants

**Figure 1:**
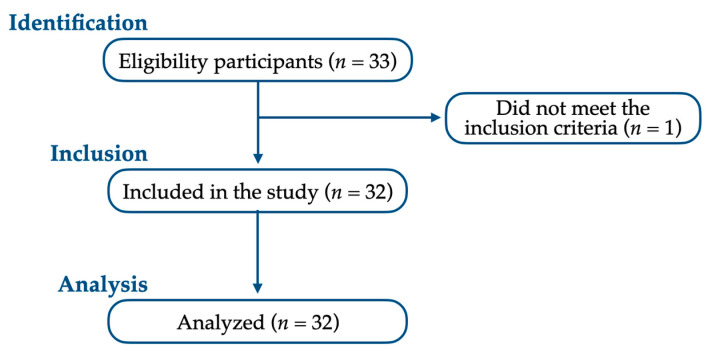
Strengthening the reporting of observational studies in epidemiology flow chart.

Table 1 presents the general characteristics of the enrolled participants, who were predominantly young (aged 28.77 ± 5.45 years) and healthy adults. Figure 1 depicts the flow of our study, which was based on the STROBE guidelines. Of the 33 potential participants obtained as the sample size, 1 participant was excluded because the eligibility requirements were not fulfilled. Consequently, 32 participants were finally enrolled in this study.

### 3.2. Jump Height Comparison

**Figure 2:**
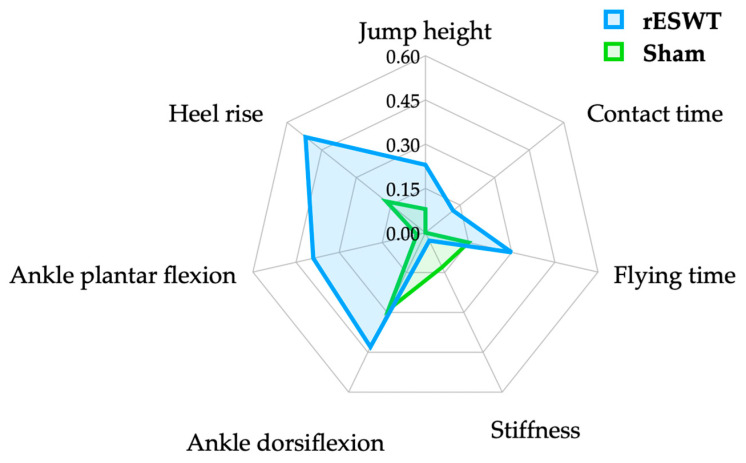
Effect size Rader chart.

In this study, the outcomes of the single-leg vertical jump, including the jump height, contact time, and flying time, were closely monitored (Figure 2 and Table 2). The rESWT group experienced a statistically significant improvement in jump height, with a small effect size (*d* = 0.23, *p* < 0.001), whereas the sham group did not show such changes. Similarly, the differences in jump height before and after the intervention were significant, with a moderate effect size (*d* = 0.55, *p* < 0.05). For flying time, there was a notable improvement in the rESWT group, as evidenced by the small effect size (*d* = 0.30, *p* < 0.001), with no change in the sham group. The impact of the intervention on flying time before and after the intervention was significant (*d* = 0.48, *p* < 0.05); however, there were no significant alterations in contact time for any group.

### 3.3. Comparison of Achilles Tendon Stiffness

In the evaluation of Achilles tendon stiffness, no statistically significant changes were detected within or between the groups. The calculated effect size was small (*d* = 0.13), with a *p*-value > 0.05, suggesting that the interventions had a minimal impact on tendon stiffness, and any observed changes were likely due to chance rather than the treatment (Figure 2 and Table 2).

### 3.4. Comparison of Ankle Joint Range of Motion

The range of motion of the ankle joint was measured in terms of dorsiflexion and plantar flexion (Figure 2 and Table 2). For dorsiflexion, both the rESWT (*d* = 0.43, *p* < 0.001) and sham (*d* = 0.29, *p* < 0.05) groups demonstrated significant improvements, although no significant differences were observed in the changes before and after the treatment (*d* = 0.34, *p* > 0.05). However, significant improvements in plantar flexion were observed only in the rESWT group (*d* = 0.39, *p* < 0.05), along with significant differences in pre- and post-treatment changes (*d* = 0.54, *p* < 0.05).

### 3.5. Heel-Rise Comparison

In the assessment phase focusing on the heel-rise test, as detailed in Figure 2 and Table 2, performance was notably enhanced post-intervention in both the rESWT group, which showed a moderate effect size (*d* = 0.52, *p* < 0.001), and the sham group, which showed a smaller yet significant effect size (*d* = 0.17, *p* < 0.05). Comparative analysis of the data collected before and after the interventions underscored these significant improvements, with the rESWT group displaying a moderate effect size (*d* = 0.73, *p* < 0.01), indicating a robust response to the treatment.

## 4. Discussion

This study investigated the impact of rESWT on activities that require robust Achilles tendon functionality, encompassing movements such as sharp direction changes, quick push-offs, and frequent jumping. Diverging from prior studies that have largely centered on pain alleviation [15,17], this study sets itself apart by targeting rESWT at Achilles tendons that demonstrated lower jump heights in a preliminary single-leg vertical jump test, and contrasting the immediate effects with those observed following a sham ESWT applied to tendons with relatively superior jump heights.

In assessing performance via the single-leg vertical jump, the rESWT group did not exhibit significant differences in contact time when compared to the sham group, although the effect size was modest. Nonetheless, notable increases in both jump height and flying time were recorded, underscoring meaningful distinctions compared to the sham group. The enhancements in the single-leg vertical jump performance, marked by an increase in jump height, indicate the potential of rESWT to bolster power generation, which is further supported by a reduction in contact time and an increase in flying time. This resonates with observations from plantar fasciitis studies, where rESWT ameliorated leg fatigue and expedited walking pace [37]. The prompt surge in muscle strength previously observed in rESWT applications to the rotator cuff may explain these results [19].

Conversely, our investigation did not corroborate a significant increase in muscle stiffness, an outcome anticipated considering jump performance enhancement. Although muscle stiffness is typically positively related to contractile force and muscle activity [37], findings from studies on patients with Parkinson’s disease suggest that an increase in muscle stiffness is not universally beneficial [38]. This highlights the necessity for further studies to unravel the intricate mechanistic significance of these observations.

Our findings indicate that both the range of motion of the ankle joint and heel-rise capacity were significantly enhanced in the sham group, albeit with a relatively diminutive effect size for dorsiflexion and heel-rise. However, the rESWT group manifested significant advancements with small-to-moderate effect sizes in dorsiflexion, plantar flexion, and heel-rise. When comparing the extent of changes across groups, the rESWT group presented more pronounced moderate effect sizes for plantar flexion (*d* = 0.54) and heel-rise (*d* = 0.73), suggesting that rESWT may be particularly effective in improving functionalities that are dependent on the strength and flexibility of the Achilles tendon.

Our results showed significant improvements in the ankle joint range of motion and heel-rise tests. Notably, the rESWT group demonstrated increases in dorsiflexion, plantar flexion, and heel-rise performance with small-to-moderate effect sizes, which are key indicators of a healthy and functional Achilles tendon. The observed improvements in the heel-rise test are consistent with the understanding that a healthy Achilles tendon is associated with a higher capacity for this movement [39]. This is relevant because the heel-rise test evaluates not only the strength and endurance of the calf muscles but also the functional integrity of the Achilles tendon itself.

Our findings resonate with those of previous studies on patients with chronic plantar fasciitis, where rESWT was found to offer benefits similar to stretching exercises. This therapy contributed to pain reduction, increased range of motion, and enhanced muscle strength during plantar flexion [40]. Since decreased ankle mobility can increase hamstring injury, ensuring ankle mobility can reduce lower limb injury [41]. This suggests that rESWT could be a valuable addition to rehabilitation protocols for various lower limb conditions, offering a noninvasive option for pain management and functional improvement.

In the context of neurological conditions, such as stroke, rESWT has been shown to positively affect spasticity, contributing to an increase in range of motion [42]. Although our study did not directly assess spasticity, improvements in plantar flexion and dorsiflexion imply that rESWT could potentially modulate neuromuscular properties that benefit patients with neurological impairments [43]. Furthermore, studies involving patients after Achilles tendon rupture repair indicated that interventions can enhance flexibility and, consequently, range of motion [44]. Our study extends this understanding by demonstrating that rESWT can significantly improve functional performance metrics, such as heel-rise, which are crucial for effective post-injury rehabilitation. Taken together, these findings suggest that rESWT is an effective modality for improving ankle joint range of motion and functionality in both pathological and postsurgical conditions. Evidence indicates its role in enhancing the mechanical and neuromuscular properties of the lower limb, which can be leveraged to optimize recovery processes and augment physical therapy outcomes.

It is worth noting that although significant improvements were observed in the rESWT group, moderate effect sizes, especially in plantar flexion (*d* = 0.54) and heel-rise (*d* = 0.73), indicate a potential clinically relevant benefit that warrants further investigation. Future studies with larger sample sizes and longitudinal designs are needed to confirm the long-term efficacy of rESWT, and to establish protocols that maximize its therapeutic effects across different patient populations.

The results of this study, which indicated significant improvements with rESWT, particularly in plantar flexion and heel-rise, suggest that rESWT could play a crucial role in improving the mechanical and neuromuscular properties of the lower limbs. These properties are essential for a broad spectrum of activities, ranging from daily movements to athletic performance. The moderate effect sizes observed advocate the inclusion of rESWT in rehabilitation programs, with further studies needed to solidify its long-term benefits and optimize therapeutic protocols. Future studies should focus on larger cohorts and extended follow-up periods to fully ascertain rESWT’s utility of rESWT in clinical practice. These findings may inform clinical decisions and encourage the integration of rESWT into treatment regimens for patients with Achilles tendon-related dysfunctions.

The limitations of this study include its short-term follow-up and small sample size, which may not capture the long-term effects of rESWT or represent a broader population. Additionally, the absence of direct measurements of muscle and tendon properties, such as sonography or MRI, indicated that changes in the structural attributes of the Achilles tendon were not directly observed. Future studies should address these limitations by including a larger cohort of participants and using a longitudinal design to observe long-term outcomes. It would also be beneficial to incorporate objective imaging techniques to correlate clinical improvements with changes in the structure of the Achilles tendon. Additionally, further studies should explore the effects of rESWT in different populations, including athletes and those with chronic Achilles tendinopathy, to broaden our understanding of its efficacy across various contexts.

## 5. Conclusions

This study suggests that rESWT has a promising role in enhancing ankle functionality, which can be integrated into rehabilitation practices. The therapy showed benefits in improving plantar flexion and heel-rise, indicating its potential to positively affect lower limb recovery. However, further studies are necessary to fully establish the long-term efficacy and optimal application of rESWT in clinical settings, particularly with larger and more diverse sample sizes over a longer period.

## Figures and Tables

**Table 1:** General characteristics.

	Mean ± Standard Deviation
Sex (male/female)	11/21
Age (years)	28.77 ± 5.45
Height (cm)	167.77 ± 7.35
Weight (kg)	61.89 ± 10.32

**Table 2:** Within- and between-group comparisons.

		rESWT	Sham	Change ^‡^
Jump height ^a^ (cm)	Pre-test	7.33 ± 4.09	8.13 ± 4.11	
	Post-test	8.32 ± 4.40	8.46 ± 4.60	−0.14 (−0.61, 0.32) ^†^
	Change ^‡^	0.23 (0.61, 1.37) ***	0.08 (−0.11, 1.28)	0.55 (0.68, 1.32) *
Contact time ^b^ (ms)	Pre-test	0.47 ± 0.17	0.43 ± 0.14	
	Post-test	0.45 ± 0.17	0.43 ± 0.14	0.01 (−0.01, 0.04) ^†^
	Change ^‡^	0.12 (−0.05, 0.01)	0.00 (−0.02, 0.02)	0.26 (−0.02, 0.01)
Flying time ^a^ (ms)	Pre-test	0.21 ± 0.07	0.23 ± 0.06	
	Post-test	0.23 ± 0.07	0.24 ± 0.07	0.00 (−0.01, 0.00) ^†^
	Change ^‡^	0.30 (0.01, 0.03) ***	0.15 (0.00, 0.02)	0.48 (0.01, 0.02) *
Stiffness ^a^ (N/m)	Pre-test	974.72 ± 199.44	963.25 ± 188.40	
	Post-test	981.94 ± 270.99	992.81 ± 268.34	−10.88 (−81.35, 59.60) ^†^
	Change ^‡^	0.03 (−52.48, 66.92)	0.13 (−27.06, 86.18)	0.13 (−25.06, 58.49)
Ankle dorsiflexion ^b^ (°)	Pre-test	12.78 ± 7.79	11.56 ± 6.81	
	Post-test	16.03 ± 7.33	13.38 ± 5.79	2.66 (0.90, 4.41) ^†^
	Change ^‡^	0.43 (1.84, 4.66) ***	0.29 (0.30, 3.32) *	0.34 (1.46, 3.66)
Ankle plantar flexion ^b^ (°)	Pre-test	59.59 ± 8.86	62.75 ± 10.59	
	Post-test	63.78 ± 12.23	62.41 ± 10.24	1.38 (−1.23, 3.98) ^†^
	Change ^‡^	0.39 (1.23, 7.15) *	0.03 (−3.14, 2.46)	0.54 (4.40, 8.53) *
Heel-rise ^a^ (cm)	Pre-test	12.01 ± 1.33	12.03 ± 1.52	
	Post-test	12.72 ± 1.39	12.28 ± 1.36	0.44 (0.20, 0.69) ^†^
	Change ^‡^	0.52 (0.50, 0.92) ***	0.17 (0.02, 0.48) *	0.73 (0.46, 0.79) **

Values are presented as mean ± standard deviation. rESWT, radial extracorporeal shock wave therapy; ^a^ parametric test; ^b^ non-parametric test; ^†^ mean change (95% confidence interval); ^‡^ Cohen’s d (95% confidence interval); * *p* < 0.05, ** *p* < 0.01, *** *p* < 0.001.

## Data Availability

Data are contained within the article.
